# Prevalence of Keratoconus in Persons With Down Syndrome in a National Registry in Norway

**DOI:** 10.1001/jamanetworkopen.2021.0814

**Published:** 2021-03-05

**Authors:** Olav Kristianslund, Liv Drolsum

**Affiliations:** 1 Department of Ophthalmology, Oslo University Hospital, Oslo, Norway; 2 Institute of Clinical Medicine, University of Oslo, Oslo, Norway

## Abstract

This cross-sectional study evaluates the association of keratoconus with Down syndrome by estimation of the prevalence of keratoconus in persons with Down syndrome in Norway.

## Introduction

Keratoconus is an eye condition with distinctive corneal characteristics, including thinning and a conical shape of the cornea. Keratoconus was previously considered rare; however, in recent studies, a prevalence of 0.2% to 0.3% has been reported^1^ and, in some studies, an even higher prevalence. An association of keratoconus with Down syndrome has been shown,^2,3^ although the exact reason remains unknown. In some studies, investigators have reported frequencies of keratoconus in persons with Down syndrome of 0% to 71%^2,3,4,5^; however, most have been limited by small sample sizes and biased patient selection. The aim of the present study was to estimate the prevalence of keratoconus among persons with Down syndrome in Norway.

## Methods

In this cross-sectional study, data were obtained from the Norwegian Patient Registry, which gathers information from all publicly funded specialist care provided in Norway. All patients who received the *International Classification of Diseases and Related Health Problems, Tenth Revision* diagnosis codes Q90 for Down syndrome and H18.6 for keratoconus from January 1, 2010, to December 31, 2019, were included. The prevalence is presented as per 100 000 population, with 95% CIs, which were calculated using SPSS, version 26 (IBM Corp). The ethical committee and data protection officer of Oslo University Hospital determined waiver of further review, study approval, and informed patient consent because the data were anonymous and reported on an aggregated group level. The research adhered to the Declaration of Helsinki. This study followed the Strengthening the Reporting of Observational Studies in Epidemiology (STROBE) reporting guideline.

## Results

Of the 238 patients with Down syndrome who had keratoconus, 122 (51.3%) were men, and 116 (48.7%) were women; the mean age was 37.1 years. A total of 4342 persons were registered as having Down syndrome in Norway during the study period. This corresponded to 0.08% of the population of Norway in the same period (mean [SD], 5 118 664 [161 758] inhabitants). Among the persons with Down syndrome, 238 (5.5%) had keratoconus, yielding an estimated prevalence of 5481 per 100 000 population (95% CI, 4834-6188 per 100 000 population).

## Discussion

An association has previously been shown between Down syndrome and keratoconus.^2,3,4^ In the present study, we estimated a prevalence of keratoconus of 5.5% among persons with Down syndrome. This prevalence is 30 times higher than that in the general population of Norway (192.1 per 100 000 population),^1^ suggesting that screening for keratoconus in these individuals should be considered to detect candidates for corneal collagen cross-linking treatment at an early stage.

Knowledge is limited concerning the cause of keratoconus and also concerning its association with Down syndrome.^2,3^ One proposed link is a collagen-related abnormality; in addition, some research has suggested that Down syndrome is associated with greater frequency of eye rubbing and possibly with more atopy.^2,4,6^ Other researchers have speculated about whether keratoconus is linked to chromosome 21.^6^

Few previous studies of keratoconus in persons with Down syndrome have been carried out in Northern Europe. A case-control study by Haugen et al^2^ revealed a thinner cornea, higher keratometry values, and a greater incidence of keratoconus in a group of participants with Down syndrome; 5 of 47 individuals with Down syndrome (10.6%) had keratoconus compared with 1 of 51 control participants (2.0%). In studies from various regions, proportions lower than those that we found have been reported in young patients, whereas higher proportions have often been reported in selected hospital populations.

To our knowledge, no previous study has reported a national prevalence of keratoconus in persons with Down syndrome. The present study used data from a nationwide patient registry in Norway, thereby avoiding the risk of selection bias. Nevertheless, no routine screening is performed for this condition, and it is reasonable to suspect that a substantial number of cases are undiagnosed, which represents a limitation of the present study in addition to some uncertainty regarding diagnosis registration.
